# Rapid Release Polymeric Fibers for Inhibition of *Porphyromonas gingivalis* Adherence to *Streptococcus gordonii*

**DOI:** 10.3389/fchem.2019.00926

**Published:** 2020-01-21

**Authors:** Mohamed Y. Mahmoud, Sonali Sapare, Keegan C. Curry, Donald R. Demuth, Jill M. Steinbach-Rankins

**Affiliations:** ^1^Department of Pharmacology and Toxicology, University of Louisville School of Medicine, Louisville, KY, United States; ^2^Center for Predictive Medicine, University of Louisville, Louisville, KY, United States; ^3^Department of Toxicology, Forensic Medicine and Veterinary Regulations, Faculty of Veterinary Medicine, Cairo University, Giza, Egypt; ^4^Department of Oral Immunology and Infectious Diseases, University of Louisville School of Dentistry, Louisville, KY, United States; ^5^Department of Microbiology and Immunology, University of Louisville School of Medicine, Louisville, KY, United States; ^6^Department of Biology, University of Louisville, Louisville, KY, United States; ^7^Department of Bioengineering, University of Louisville Speed School of Engineering, Louisville, KY, United States

**Keywords:** periodontal disease, *Porphyromonas gingivalis*, *Streptococcus gordonii*, oral biofilm, electrospun fibers, peptide delivery

## Abstract

Active agents targeting key bacterial interactions that initiate biofilm formation in the oral cavity, may alter periodontitis progression; however, to date, specifically-targeted prophylactic and treatment strategies have been limited. Previously we developed a peptide, BAR (SspB Adherence Region), that inhibits oral *P. gingivalis/S. gordonii* biofilm formation *in vitro* and *in vivo*, and BAR nanoparticles that increase BAR effectiveness via multivalency and prolonged delivery. However, limited BAR loading and nanoparticle retention in the oral cavity can result in inadequate release and efficaciousness. Given this, an effective delivery platform that can release concentrations of BAR suitable for twice-daily applications, may offer an alternative that enhances loading, ease of administration, and retention in the oral cavity. With this in mind, the study objectives were to develop and characterize a rapid-release platform, composed of polymeric electrospun fibers (EFs) that encapsulate BAR, and to evaluate fiber safety and functionality against *P. gingivalis*/*S. gordonii* biofilms *in vitro*. Poly(lactic-co-glycolic acid) (PLGA), poly(L-lactic acid) (PLLA), and polycaprolactone (PCL) were electrospun alone or blended with polyethylene oxide (PEO), to provide high BAR loading and rapid-release. The most promising formulation, 10:90 PLGA:PEO EFs, provided 95% BAR release after 4 h, dose-dependent inhibition of biofilm formation (IC50 = 1.3 μM), disruption of established dual-species biofilms (IC50 = 2 μM), and maintained high cell viability. These results suggest that BAR-incorporated EFs may provide a safe and specifically-targeted rapid-release platform to inhibit and disrupt dual-species biofilms, that we envision may be applied twice-daily to exert prophylactic effect in the oral cavity.

## Introduction

Periodontal disease is a group of chronic inflammatory diseases that are globally prevalent, affecting over 65 million adults in the U.S., with increased incidence in developing countries. Moreover, the prevalence and severity of periodontal disease has been shown to increase from 47 to 64% in adults from age 30–65 (Eke et al., 2015). Advanced periodontal disease (subgingival pocket depths > 6 mm) occurs in up to 11% of adults worldwide (Kassebaum et al., 2014), and is a chronic, irreversible inflammatory disease that results in destruction of connective tissue, vascular proliferation, and alveolar bone resorption (Pihlstrom et al., 2005). *Porphyromonas gingivalis* is strongly associated with chronic adult periodontitis (Socransky et al., 1998; Darveau et al., 2012; Griffen et al., 2012) and has been considered to be a key pathogen that may promote disease by perturbing host-microbe homeostasis, leading to uncontrolled inflammation (Darveau et al., 2012). While the primary niche of *P. gingivalis* is the anaerobic environment of the subgingival pocket, *P. gingivalis* initially colonizes the oral cavity by interacting with Gram-positive commensal streptococci in the supragingival environment (Marsh, 1994). These initial adhesive interactions thus represent ideal points for intervention to prevent *P. gingivalis* colonization and can be targeted with specifically designed biologics that may effectively curtail the progression of periodontal disease (Daep et al., 2006).

Previous work in our groups has shown that the adherence of *P. gingivalis* with commensal oral streptococci such as *S. gordonii*, is mediated by the interaction of the minor fimbrial antigen, Mfa1, of *P. gingivalis* with the streptococcal antigen I/II protein (e.g., SspB). We also showed that a discrete region, designated BAR (SspB Adherence Region), is essential to adherence (Brooks et al., 1997). A synthetic peptide comprised of amino acids 1167–1193 from this region potently inhibited adherence of *P. gingivalis* with *S. gordonii* (IC50 = 1.3 μM) (Daep et al., 2006), and significantly reduced *P. gingivalis* virulence in a mouse model of periodontitis (Daep et al., 2011). However, BAR peptide exhibited weaker and more transient effectiveness against pre-established dual-species biofilms and more complex biofilms. In addition, alternative non-targeted prophylactic therapies including scaling and root planning have only been temporarily effective in removing the subgingival biofilm and halting the corresponding inflammatory cascade (Herrera et al., 2012), since the biofilm begins to re-form shortly after prophylaxis is completed. Furthermore, while current medicinal therapies, consisting of systemic and local antibiotic administration, are initially effective, they can result in side effects due to an inadequate concentration of drug reaching the periodontal pockets, corresponding transient activity (Drisko, 1996; Walker, 1996; Allaker and Ian Douglas, 2015), and the development of antimicrobial resistance. Moreover, the non-specific nature of current antibiotic agents can adversely impact the commensal microbial community. Given these challenges, new prophylactic and therapeutic approaches that provide more specific targeting of periodontal pathogen interactions are urgently needed to address these shortcomings and to improve oral therapeutic outcomes.

Delivery vehicles that localize the delivery and maintain the stability of specifically-targeted biologics, such as BAR peptide, may offer improved functional activity, thereby enhancing the therapeutic efficacy (Garg et al., 2012)_._ Delivery platforms such as electrospun fibers (EFs) have been used in a variety of applications like wound dressing (Liu et al., 2017), tissue regeneration (Inanç et al., 2009; Yang et al., 2009), and antimicrobial delivery (Reise et al., 2012; Chaturvedi et al., 2013) to incorporate water-soluble bioactive agents such as proteins, peptides, nucleic acids and hydrophilic/hydrophobic drugs. Polymeric fibers can protect encapsulated cargo from premature degradation, in addition to minimizing systemic absorption and associated side effects. Moreover, electrospinning offers a cost-effective, reproducible, and highly tunable method to provide efficient encapsulation and release based on the needs of rapid-onset or prolonged delivery applications. Many studies have shown that fibers composed of natural, synthetic, and semi-synthetic polymers and polymer blends can tune drug miscibility and that the resulting drug-polymer interactions may lead to different release profiles (Chou and Woodrow, 2017).

We previously showed that BAR-modified and BAR-encapsulated nanoparticles inhibit *P. gingivalis* biofilm formation (Kalia et al., 2017; Mahmoud et al., 2018, 2019). These vehicles were envisioned to serve in formulations such as an oral gel, varnish or mouthwash that require two to three daily applications. Here we sought to develop and characterize EFs that may be administered in future applications, as rapid-release dental strips in the oral cavity. We proposed that the development of an effective oral delivery system that can release BAR within a time frame desired for twice-daily applications, may offer an alternative platform that increases loading, facilitates ease of administration, and provides the potential of enhanced retention in the oral cavity. Since biocompatible, biodegradable, and Food and Drug Administration (FDA) approved polymers including poly(lactic-co-glycolic) acid (PLGA) (Li et al., 2002), poly(L-lactic acid) (PLLA) (Jun et al., 2003), polycaprolactone (PCL) (Chaturvedi et al., 2013), and polyethylene oxide (PEO) (Son et al., 2004) have been successfully electrospun and used in clinical applications, we hypothesized that EFs comprised of these polymers may offer advantages to BAR peptide administration in the oral cavity. To obtain maximal delivery within our time frame of interest (e.g., twice-daily), we hypothesized that BAR release may be modulated by changing the hydrophobic:hydrophilic polymer ratios of the blended fibers.

Given this, the goal of this work was to synthesize, characterize, and demonstrate the preliminary inhibitory and disruptive capabilities of non-blended and blended EF formulations to prevent and treat *P. gingivalis*/*S. gordonii* biofilm formation. We demonstrated that changing the hydrophobic:hydrophilic polymer ratios altered the release kinetics of BAR peptide for durations relevant to oral application. Moreover, we functionally characterized the effectiveness of EFs in preventing the formation of *P. gingivalis*/*S. gordonii* biofilms *in vitro*. These results suggest that BAR-incorporated EFs can be formulated to release peptide over a time frame of hours and may represent a new dosage form that can release targeting molecules in the oral cavity. Long-term, we envision that BAR-EFs may provide a promising rapid-release platform to deliver BAR peptide to the oral cavity in the form of strips or gum that can be conveniently applied twice-daily to inhibit biofilm formation.

## Materials and Methods

### Materials

Hydrophobic polymers including poly(lactic-co-glycolic acid) (PLGA, 50:50 lactic:glycolic acid, MW 30,000–60,000), poly(L-lactic acid) (PLLA, MW 50,000), and polycaprolactone (PCL, MW 80,000), and the hydrophilic polymer, polyethylene oxide (PEO, MW 100,000) were purchased from Sigma-Aldrich (St. Louis, MO, USA). Tris-EDTA (TE) buffer (pH 8.0), phosphate buffered saline (PBS), and the organic solvents chloroform, dimethyl sulfoxide (DMSO), and hexafluoroisopropanol (HFIP) were also purchased from Sigma-Aldrich (St. Louis, MO, USA). All chemicals were used directly without further purification. One milliliter plastic syringes, petri dishes, and 20 mL scintillation vials were obtained from VWR. One milliliter glass syringes were purchased from Fisher Scientific. The electrospinner was provided courtesy of Dr. Stuart Williams at the Cardiovascular Innovative Institute, University of Louisville.

### Peptide Synthesis

The peptide used in this study (NH_2_-LEAAPKKVQDLLKKANITVKGAFQLFS-COOH) (Daep et al., 2008) was synthesized by BioSynthesis, Inc. (Lewisville, TX). It was obtained with purity <94% and comprised residues 1167–1193 of the SspB (Antigen I/II) protein sequence of *S. gordonii*. A fluorescent BAR peptide (F-BAR), synthesized by covalently attaching 6-carboxyfluorescein (F-BAR) to the epsilon amine of the lysine residue underlined in the sequence above, was used to more easily characterize BAR loading and release from the fibers via fluorescence detection (Kalia et al., 2017).

### Preparation of Polymer Solutions

To prepare the hydrophobic-only (non-blended) polymer fiber batches, PLGA and PLLA were dissolved in HFIP at a concentration of 15% (w/w), while PCL was dissolved in HFIP at a concentration of 12% w/w due to increased viscosity. The polymer solutions were aspirated into a 7 mL glass scintillation vials, and sealed using aluminum foil and parafilm to prevent evaporation of the organic solvent. The vials were placed in a shaker at 150 rpm and incubated at 37°C overnight to solubilize the polymer. The final volume of each polymer solution was 1 mL. The following day, F-BAR peptide was dissolved in 200 μL TE buffer and mixed with the polymer solvents at a concentration of 1% w/w (e.g., 2.4 mg F-BAR/240 mg polymer).

To prepare blended polymers, the hydrophobic polymers PLGA, PLLA, and PCL were mixed with PEO at different ratios (40:60, 20:80, 10:90 w/w) to form PLGA:PEO, PLLA:PEO, and PCL:PEO blends in chloroform at a concentration of 15% (w/v). The blended solutions were aspirated into 20 mL glass scintillation vials, and sealed using parafilm to prevent evaporation of the organic solvent. The vials were placed in a shaker at 150 rpm and incubated at 37°C overnight to solubilize the polymer. The final volume of each polymer solution was 1 mL. The following day, F-BAR peptide was dissolved in 60 μL DMSO. The F-BAR solutions were mixed with the polymer solvent at a concentration of 1% w/w (BAR/polymer content) (Kim et al., 2007).

### Electrospinning

For the non-blended polymer solutions, 1 mL of the mixed polymer suspension was aspirated into a 1 mL plastic syringe with an 18-gauge blunt needle tip. The internal diameter of the BD plastic syringe (4.78 mm), was set in the syringe pump program. The collector was adjusted such that there was at least 10 cm distance maintained from the needle tip. The syringe pump motor controls were adjusted by setting the “slide” control to 4.5 and the “rotor” to 8. The voltage supply was set at 20 kV, and the syringe pump flow rate was set to 0.8 mL per hour. The polymer solution was electrospun at room temperature, under atmospheric conditions, for 1 h 15 min, and the resulting fine mist was collected on the mandrel and allowed to dry for 15 min. The mandrel was removed from the collector and the fiber was cut and gently peeled off the mandrel. The fiber was placed in a labeled petri dish and kept in a desiccator for 24 h before characterization. The desiccated fibers were stored in 4°C until use (Tyo et al., 2017).

For the blended polymer solutions, 1 mL of the mixed dual-polymer suspension was aspirated into a 1 mL glass syringe with a 22-gauge blunt needle tip. The internal diameter of the Hamilton gastight syringe (4.61 mm), was set in the syringe pump program. A distance of 15 cm was kept between the needle tip and the collector. The “slide” control was set to 4.5 and the “rotor” control was set to 8. A voltage of 20-25 kV was applied, at a flow rate of 0.3 mL per hour. The electrospinning processes were employed under ambient conditions for 3 h 20 min. The stretched and solidified polymeric fibers were collected on a 4 mm diameter stainless steel mandrel and allowed to dry for 15 min. Similar desiccation and storage conditions were followed, as noted for the non-blended fibers.

### EF Characterization: EF Morphology, Diameter, BAR Loading, and Release

Fiber morphology and size were evaluated using scanning electron microscopy (SEM) (JSM-820, JEOL, Tokyo, Japan), and fiber diameters were obtained by analyzing SEM images with NIH ImageJ. The loading and encapsulation efficiency (EE) of F-BAR peptide in the non-blended and blended fibers were determined by dissolving F-BAR fibers in DMSO. The fiber solution was subsequently vortexed, sonicated for 5 min, and dissolved for 1 h in a dark room. The quantity of extracted F-BAR was determined by measuring the fluorescence using a spectrophotometer (488/518 nm excitation/emission), relative to an F-BAR standard (Kalia et al., 2017; Mahmoud et al., 2018, 2019). A standard curve of F-BAR was obtained by adding 0.1 mg F-BAR to 1 mL of 1:9 DMSO:TE, and serially diluting in 1:9 DMSO:TE. The diluted solutions (100 μL/well) were transferred to a 96-well clear bottom microtiter plate in triplicate. For the dissolved fiber samples, after the incubation period, the fiber sample solutions were vortexed and sonicated again. The solutions were diluted 1:2, 1:5, 1:10, and 1:100 in 1:9 DMSO:TE solution, and transferred to a microtiter plate.

The *in vitro* release of F-BAR from fibers was measured by gentle agitation of EFs in phosphate buffered saline (PBS, pH 7.4) at 37°C. At fixed time points (1, 2, 4, 8, 12, and 24 h), samples were collected and the amount of F-BAR released from the EFs was quantified via fluorescence spectroscopy, against an F-BAR standard in PBS (Kalia et al., 2017; Tyo et al., 2017; Mahmoud et al., 2018).

### Growth of Bacterial Strains

*P. gingivalis* (ATCC 33277) was grown in Trypticase soy broth (Difco Laboratories Inc., Livonia, MI, USA) supplemented with 0.5% (w/v) yeast extract, 1 μg/mL menadione, and 5 μg/mL hemin. The medium was reduced for 24 h under anaerobic conditions (10% CO_2_, 10% H_2_, and 80% N_2_) and *P. gingivalis* was subsequently inoculated and grown anaerobically for 48 h at 37°C. *S. gordonii* DL-1 was cultured aerobically without shaking in brain-heart infusion broth (Difco Laboratories Inc.) supplemented with 1% yeast extract for 16 h at 37°C (Daep et al., 2006).

### Biofilm Inhibition Assay

To assess the effectiveness of BAR-incorporated EFs to prevent the interaction of *P. gingivalis* with *S. gordonii, S. gordonii* was harvested from culture and labeled with 20 μL of 5 mg/mL hexidium iodide for 15 min at room temperature. Following incubation, cells were centrifuged to remove unbound fluorescent dye. The bacterial concentration was subsequently measured by the O.D. (600 nm) from 20-fold diluted cultures of *S. gordonii*. The optical density of *S. gordonii* cells was adjusted to 0.8 O.D. (1 × 10^9^ CFU/mL) to obtain uniformity between cell counts in each well. After adjusting the optical density, 1 mL of *S. gordonii* cells was added to each well of 12-well culture plates containing a sterilized micro-coverslip. The cell culture plates were wrapped in aluminum foil to protect the labeled cells from light and placed on a rocker platform in the anaerobic chamber for 24 h.

*P. gingivalis* cultures were optimized using a similar approach, utilizing a different fluorescent label (20 μL of 4 mg/mL carboxyfluorescein–succinylester). *P. gingivalis* was incubated with the fluorescent dye for 30 min on a rocker platform and protected from light. The same procedures were followed as performed with *S. gordonii* to determine cell concentration, with slight adaptations. The optical density of *P. gingivalis* was adjusted from 0.8–0.4 O.D. (5 × 10^7^ CFU/mL) by diluting *P. gingivalis* cultures with an equal volume of 1X PBS containing BAR-EFs, free BAR, or blank EFs as a control, to a final volume 1 mL. The final concentration of BAR-EFs or free BAR ranged from 0.3–3 μM based on the previously determined IC50 of free BAR (1.3 μM). *P. gingivalis* was incubated with BAR-EFs, free BAR, or blank EFs at 25°C for 30 min before transferring to wells containing *S. gordonii*.

Plates containing *P. gingivalis* and *S. gordonii* were subsequently incubated for 24 h at 37°C in anaerobic conditions. The following day, the supernatant was removed and cells were washed with PBS. Adherent cells were fixed with 4% (w/v) paraformaldehyde and the cover glass was mounted on a glass slide. Biofilms were visualized using a Leica SP8 confocal microscope (Leica Microsystems Inc., Buffalo Grove, IL) under 60× magnification. Background noise was minimized using software provided with the Leica SP8 and three-dimensional z-stack biofilm images were obtained from 30 randomly chosen frames using a z-step size of 0.7 μm. Images were analyzed with Volocity image analysis software (version 6.3; Perkin Elmer, Waltham, MA, USA) to determine the ratio of green to red fluorescence (GR), representing *P. gingivalis* and *S. gordonii*, respectively. Control samples were used to subtract background levels of auto-fluorescence. Briefly, triplicate samples of *S. gordonii* alone were immobilized without *P. gingivalis* or BAR in 12-well culture plates and the same procedures for dual-species biofilm were followed. *S. gordonii*-only coverslips were visualized and images were analyzed as described above. The GR background was subtracted using the following formula: GR sample or control–GR *S. gordonii-*only. Each treatment group (BAR-EFs or free BAR) was analyzed in triplicate and three independent frames were measured for each well. GraphPad InStat (La Jolla, CA) was used for data analysis and differences were considered to be statistically significant when *P* ≤ 0.05. The percent inhibition of *P. gingivalis* adherence was calculated with the following formula: GR sample/GR control (Kalia et al., 2017; Mahmoud et al., 2018).

### Biofilm Disruption Assay

The same procedures utilized in the inhibition assay were followed, except *P. gingivalis* was allowed to adhere to *streptococci* in the absence of BAR peptide or BAR-EFs to demonstrate the ability of BAR-incorporated EFs to disrupt or “treat” pre-established biofilms. The resulting *P. gingivalis/S. gordonii* biofilms were then treated for the maximum duration observed for free BAR to disrupt existing biofilms (3 h) (Kalia et al., 2017). Established biofilms were administered BAR-EFs, free BAR or blank EFs at various concentrations in 1 mL PBS, and processed and analyzed as described above. The mean and standard deviation (SD) between samples were determined and the percent disruption of *P. gingivalis* adherence was calculated with the following formula: GR sample/GR control (Kalia et al., 2017; Mahmoud et al., 2018).

### Tissue Culture

Telomerase immortalized gingival keratinocytes (TIGKs) were grown on 12-well collagen-coated plates (Becton Dickinson, Bedford, MA) and cultured using DermaLife K Calcium Free Medium (LifeFactors®) supplemented with penicillin/streptomycin (100 U/mL final concentration; St. Louis, MO), insulin (5 μg/mL), recombinant human (rh), L-glutamine (6 mM), apo-transferrin (5 μg/mL), epinephrine (1 μM), rh TGF-α (0.5 ng/mL), extract P™, calcium chloride (0.06 mM) and hydrocortisone hemisuccinate (100 ng/mL). The cells were incubated at 37°C in the presence of 5% CO_2_ for 6 days until they reached 95% confluence (Mahmoud et al., 2019).

### Determination of BAR and BAR-EFs Safety *in vitro*

#### Hemolytic Assay

A sample of 250 μL of 1% sheep erythrocytes (Rockland Inc., Pennsylvania, USA) was suspended in sterile PBS. The IC50 and maximum effective concentrations (1.3 and 3.4 μM, respectively) of free BAR peptide or BAR in 10:90 PLGA:PEO BAR-EFs used in *in vitro* and *in vivo* studies, were added to sheep erythrocytes. Water replaced PBS as a positive control for cell hemolysis. The suspension was incubated at 37°C for 3 h then centrifuged at 3,500 × g. Hemoglobin released due to cell lysis was analyzed by measuring the absorbance at 541 nm (Mahmoud et al., 2019).

#### MTT Assay

TIGK cells were seeded in 12-well plates at a density of 6 × 10^4^ cells in 1 mL media per well and incubated for 24 h to allow for 60–70% confluency and sufficient adhesion. Cells were treated with 1.3 or 3.4 μM of BAR or 10:90 PLGA-PEO BAR-EFs. After 24 h, 100 μL of MTT solution was added to the media of all samples. After 4 h incubation at 37°C, 550 μL of lysis buffer was added to the media of each well and plates were incubated for overnight. The absorbance of each well was read at 570 nm, and the sample absorbance was normalized to the absorbance of medium-only treated cells. Cells were treated with 10% DMSO media (100 μL DMSO in 900 μL media) as a positive control for cell death (Tyo et al., 2017; Mahmoud et al., 2019).

#### ATP Assay

The metabolic activity of TIGK cells was assessed by measuring total ATP levels using the CellTiter-Glo reagent (Promega, Madison WI), as described by the manufacturer. TIGK cells were seeded at a density of 6 × 10^4^ cells in 1 mL media per well and incubated at 37°C, 5% CO_2_ for 24 h in a 12-well flat bottom plate. Cells were then incubated with BAR or 10:90 PLGA-PEO BAR-EFs (1.3 or 3.4 μM) for 24 h at 37°C in 5% CO_2_. Cells were then lysed with 500 μL of 0.1% Triton X-100 for 30 min at 37°C. The lysates were collected and centrifuged at 1,000 × g for 10 min at 4°C, and 50 μL of supernatant was mixed with 50 μL of CellTiter-Glo reagent. Samples were incubated at ambient temperature for 10 min in a black 96-well plate in the dark. Total luminescence was measured with a Victor 3 luminometer (Perkin-Elmer, Inc.). Cells incubated with 1 ng of staurosporine or with medium-only served as positive and negative controls for cell death, respectively (Mahmoud et al., 2019).

#### LDH Assay

Cell membrane leakage was measured by assessing the release of lactate dehydrogenase (LDH). Extracellular LDH was quantified using a CytoTox96® non-radioactive cytotoxicity assay (Promega, Madison WI) as described by the manufacturer. TIGK cells were plated at density of 6 x 10^4^ cells in 1 mL media per well in a 12-well flat bottom plate, and incubated at 37°C, 5% CO_2_ for 24 h. BAR or 10:90 PLGA-PEO BAR-EFs (1.3 or 3.4 μM) were added to cells in triplicate for 24 h at 37°C in 5% CO_2_. Fifty microliters of supernatant from free BAR and BAR-EF-treated (1.3 and 3.4 μM) cells were added to the LDH substrate and incubated at room temperature for 30 min. Then the reactions were terminated by adding 50 μL of stop solution. LDH activity was determined by measuring the optical density of the solution at 490 nm. Cells incubated with staurosporine or with medium-only served as positive and negative controls for toxicity, respectively (Mahmoud et al., 2019).

#### Oxidative DNA Damage

Free radicals and other reactive species are generated from cells under stress and cause oxidative damage to biomolecules. DNA is the most targeted site of oxidative attack. The apurinic/apyrimidine (AP or abasic) site is a prevalent oxidative DNA damage lesion. OxiSelect™ Oxidative DNA Damage Quantitation Kit (Cell Biolabs, INC., San Diego, CA, USA) was used to quantify AP sites in cells treated with free BAR or 10:90 PLGA-PEO BAR-EFs (1.3 or 3.4 μM) as described by the manufacturer. TIGK cells were plated at density of 6 x 10^4^ cells in 1 mL media per well in a 12-well flat bottom plate, and incubated at 37°C, 5% CO_2_ for 24 h. BAR or BAR-EFs (1.3 or 3.4 μM) were added to cells in triplicate for 24 h at 37°C in 5% CO_2_. Cells treated with 2 mM H_2_O_2_ or medium-only served as positive and negative controls for DNA damage, respectively. Genomic DNA was isolated from TIGK cells by QIAamp DNA Mini kit (Qiagen). AP sites were determined in genomic DNA by using a biotinylated aldehyde reactive probe (ARP) that reacts specifically with an aldehyde group of AP sites, followed by colorimetric detection using a streptavidin–enzyme conjugate (450 nm). The quantity of AP sites in DNA samples was determined by comparing the absorbance with standard curve of known amount of AP sites (Thakur et al., 2018).

### Statistical Analysis

Data from each of the toxicity tests were analyzed using ANOVA after passing Bartlett's and Brown-Forsythe tests for homogeneity of variances using GraphPad InStat (La Jolla, CA). A pair-wise, parametric analysis of variance using a Bonferroni multiple comparison *post-hoc* test was used to determine the statistical difference among the individual groups. A *P* ≤ 0.05 was considered to be statistically significant.

## Results

### EF Characterization: EF Morphology, Diameter, BAR Loading, and Release

**Figure 1:**
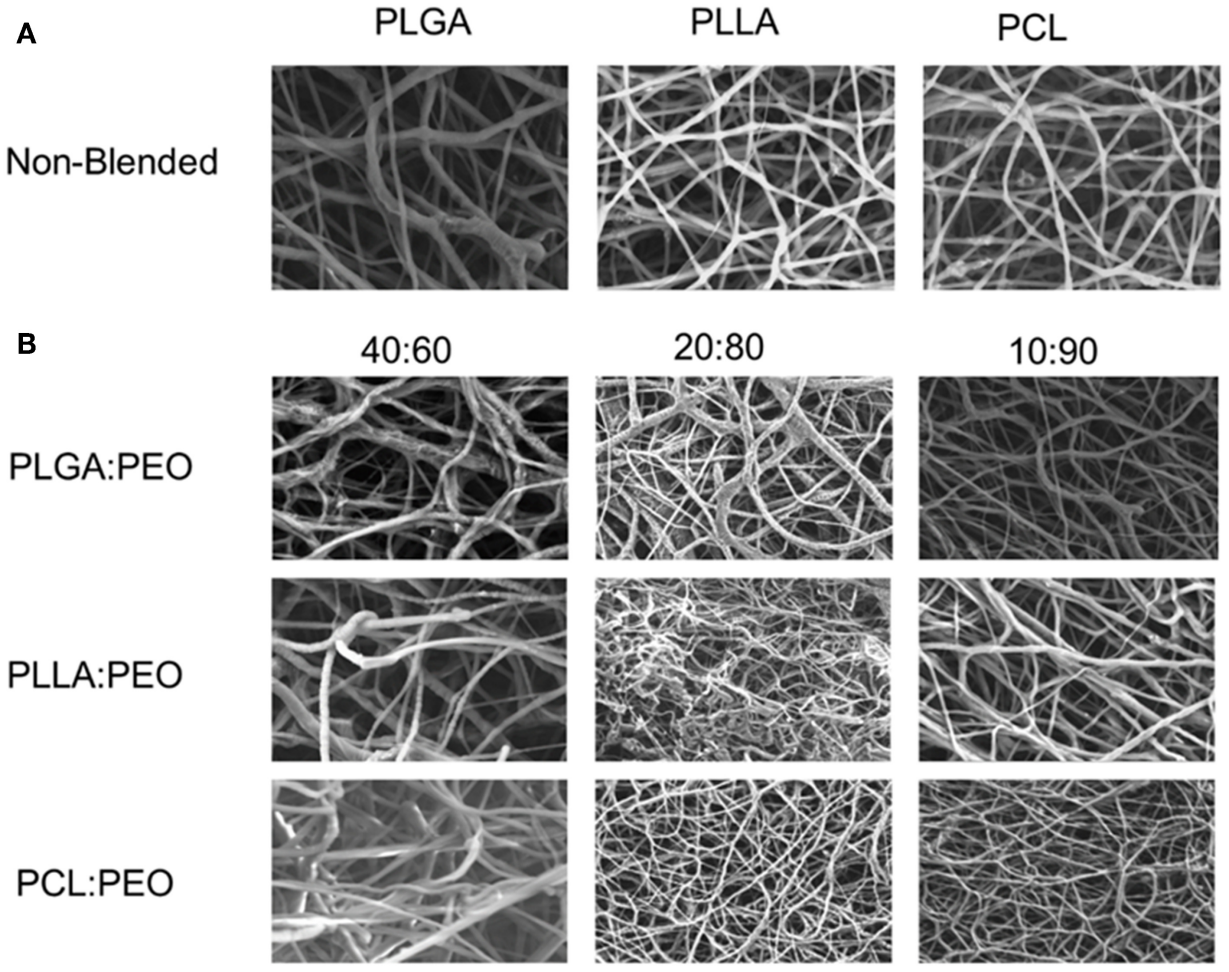
**(A)** SEM images of 1% w/w BAR PLGA, PLLA, and PCL non-blended fibers. **(B)** SEM images of 40:60, 20:80, and 10:90 1% w/w BAR blended PLGA:PEO, PLLA:PEO, and PCL:PEO fibers.

**Figure 2:**
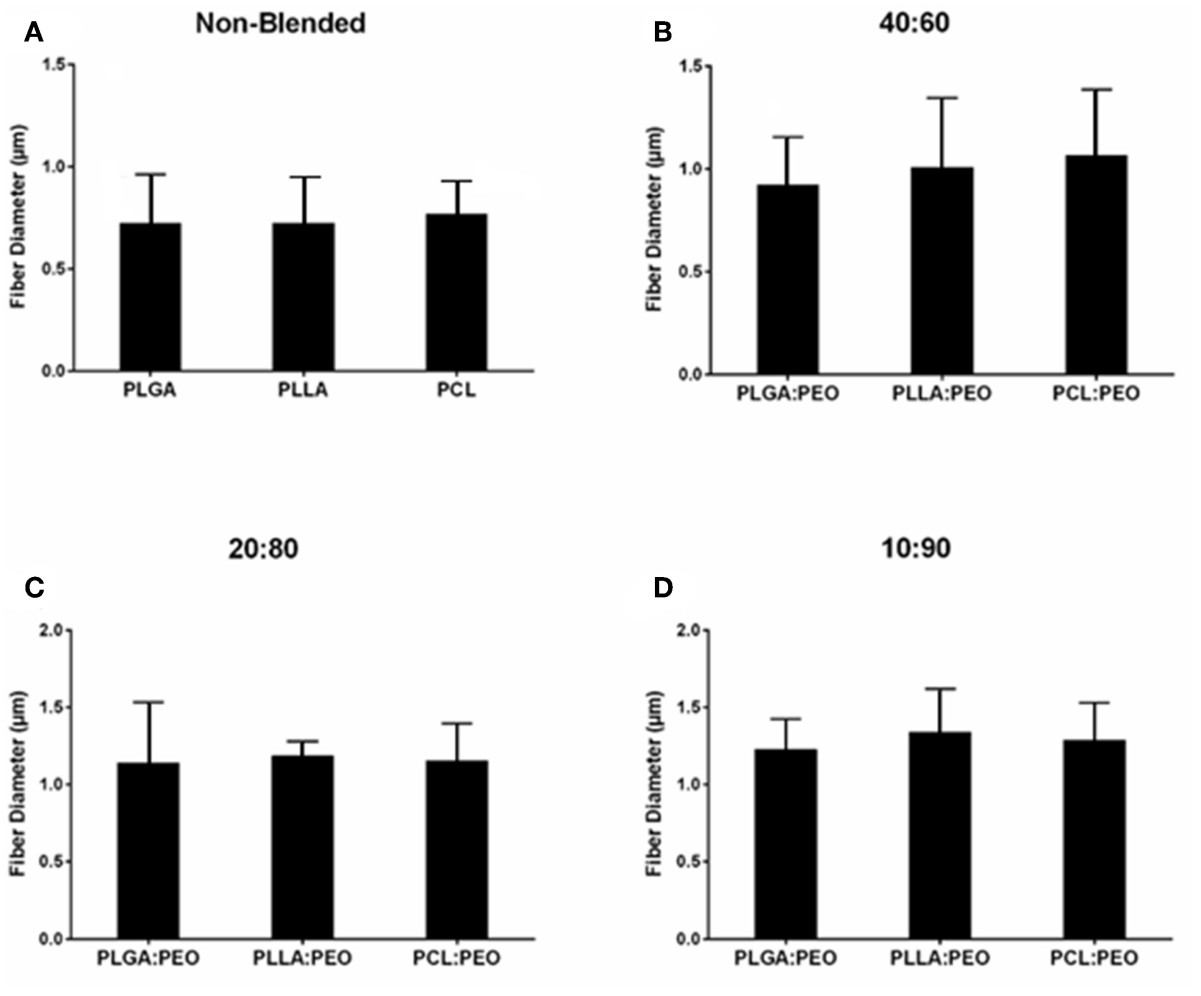
Average diameters of electrospun fibers measured from SEM images, using ImageJ. **(A)** Non-blended and blended **(B)** 40:60, **(C)** 20:80, and **(D)** 10:90 PLGA:PEO, PLLA:PEO, and PCL:PEO 1% w/w BAR fibers. Error bars represent the mean ± the standard deviation (*n* = 3) of three independent experiments.

Fiber morphologies and diameters are shown in Figures 1, 2. The average diameters of EFs ranged from 0.7 to 1.3 μm with no statistical significance observed within or across different formulations, as a function of polymer type or blend ratio.

### BAR Loading and Release

**Figure 3:**
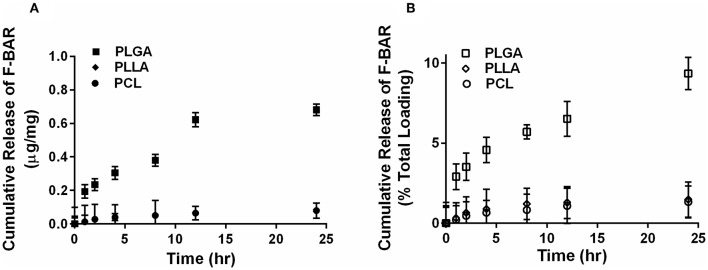
The cumulative release of F-BAR from 1% w/w F-BAR non-blended (100:0) PLGA, PLLA, and PCL fibers. The cumulative release is reported as **(A)** μg F-BAR per mg of fiber, and **(B)** percent of total loaded F-BAR. PLGA showed the greatest release of incorporated BAR among the non-blended formulations at 24 h. Error bars represent the mean ± the standard deviation (*n* = 3) of three independent experiments.

The overall polymer yield after electrospinning ranged from 40 to 60% for the non-blended fiber formulations, while the blended fibers achieved higher yields spanning 80–90%. The total F-BAR loading for non-blended and blended EFs ranged between 4.6 and 6.9 μg BAR/mg polymer and 6.0–9.2 μg BAR/mg polymer, respectively, indicating that high loading of F-BAR was achieved in all fiber formulations (Table 1). To determine the amount of F-BAR release from the different fiber formulations, F-BAR EFs were incubated in PBS at 37°C. The fluorescence of the collected supernatant was measured at 1, 2, 4, 8, 12, and 24 h. Figure 3 shows the cumulative release of F-BAR from non-blended EFs at each time point over a 24 h duration. PLGA EFs demonstrated minimal release of F-BAR (9.5% of total loading) after 24 h, while PLLA and PCL fibers showed even less release during the same duration. Overall, EFs consisting of only hydrophobic polymers (i.e., non-blended formulations) demonstrated minimal release relative to the PEO-blended EFs.

**Table 1:** The amount of BAR loaded in non-blended and blended polymeric EF formulations (μg/mg) and percent of total BAR loaded in blended and blended EFs.

**Fiber formulation**	**Blend ratio**	**Overall polymer yield(%)**	**Loading BAR/fiber (μg/mg)**	**Encapsulation efficiency (%)**
PLGA	100:0	59.0	6.9 ± 0.1	69 ± 2.5
PCL		51.0	6.0 ± 0.4	60 ± 4.0
PLLA		42.3	4.6 ± 0.6	46 ± 5.2
PLGA:PEO	40:60	82.9	7.4 ± 0.5	74 ± 5.5
PCL:PEO		91.5	8.6 ± 0.2	86 ± 2.4
PLLA:PEO		82.0	9.1 ± 0.3	92 ± 3.1
PLGA:PEO	20:80	80.9	8.8 ± 0.2	88 ± 2.6
PCL:PEO		89.3	8.9 ± 0.4	89 ± 4.0
PLLA:PEO		85.2	8.3 ± 0.4	83 ± 4.2
PLGA:PEO	10:90	82.8	8.8 ± 0.5	88 ± 5.6
PCL:PEO		80.0	6.0 ± 0.4	60 ± 4.0
PLLA:PEO		80.9	8.5 ± 0.3	85 ± 3.5

*High loading and encapsulation efficiency were achieved in all fiber formulations. However, non-blended EFs showed comparatively lower polymer yield and encapsulation efficiency, relative to the blended EFs. Data represent the mean ± standard deviation (n = 3) of three independent samples*.

**Figure 4:**
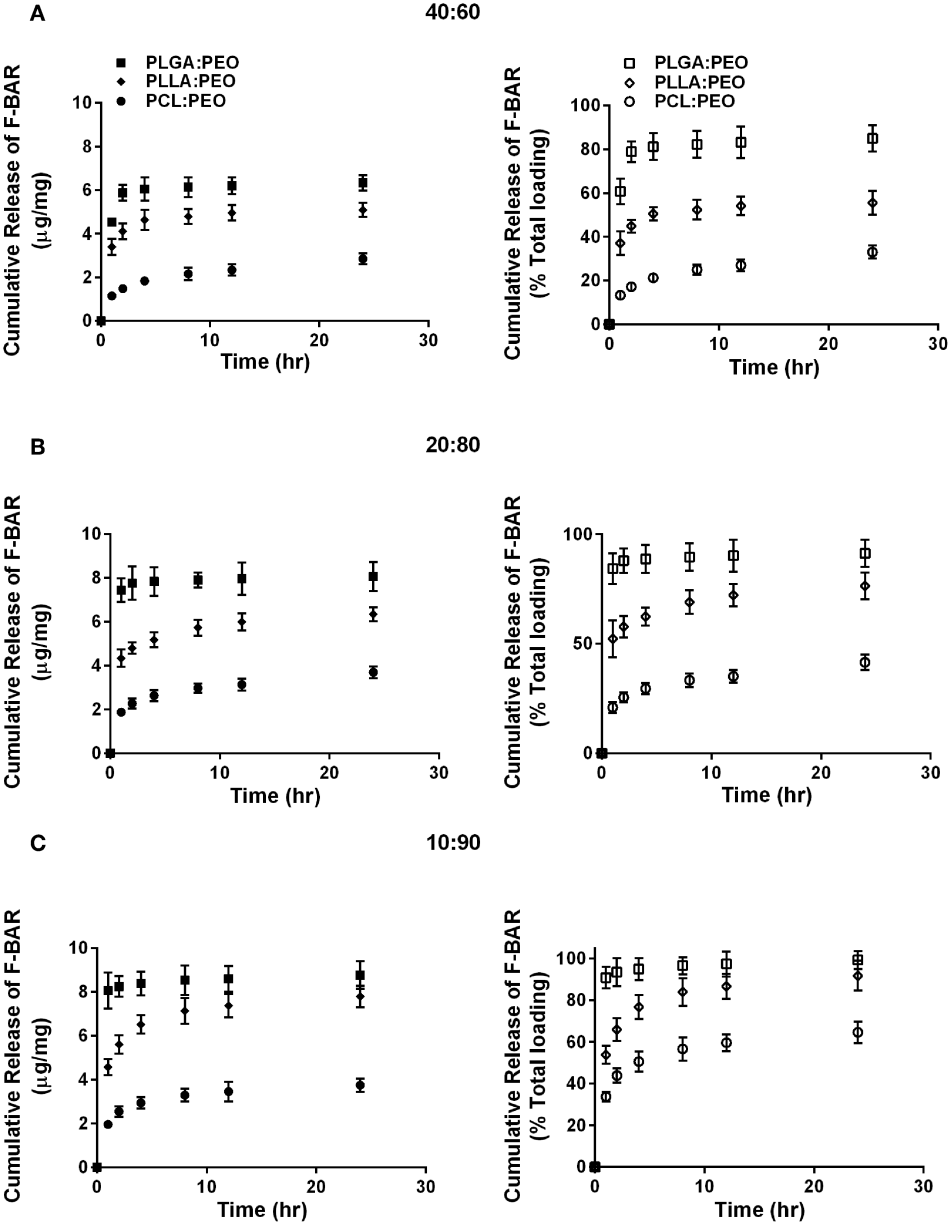
The cumulative release of F-BAR from 1% w/w F-BAR blended PLGA:PEO, PLLA:PEO, and PCL:PEO fibers **(A)** 40:60, **(B)** 20:80, and **(C)** 10:90. The cumulative release is reported as the total quantity of F-BAR released on the left (μg F-BAR per mg of fiber), and as the percent of total loaded F-BAR on the right. Error bars represent the mean ± the standard deviation (*n* = 3) of three independent experiments.

**Figure 5:**
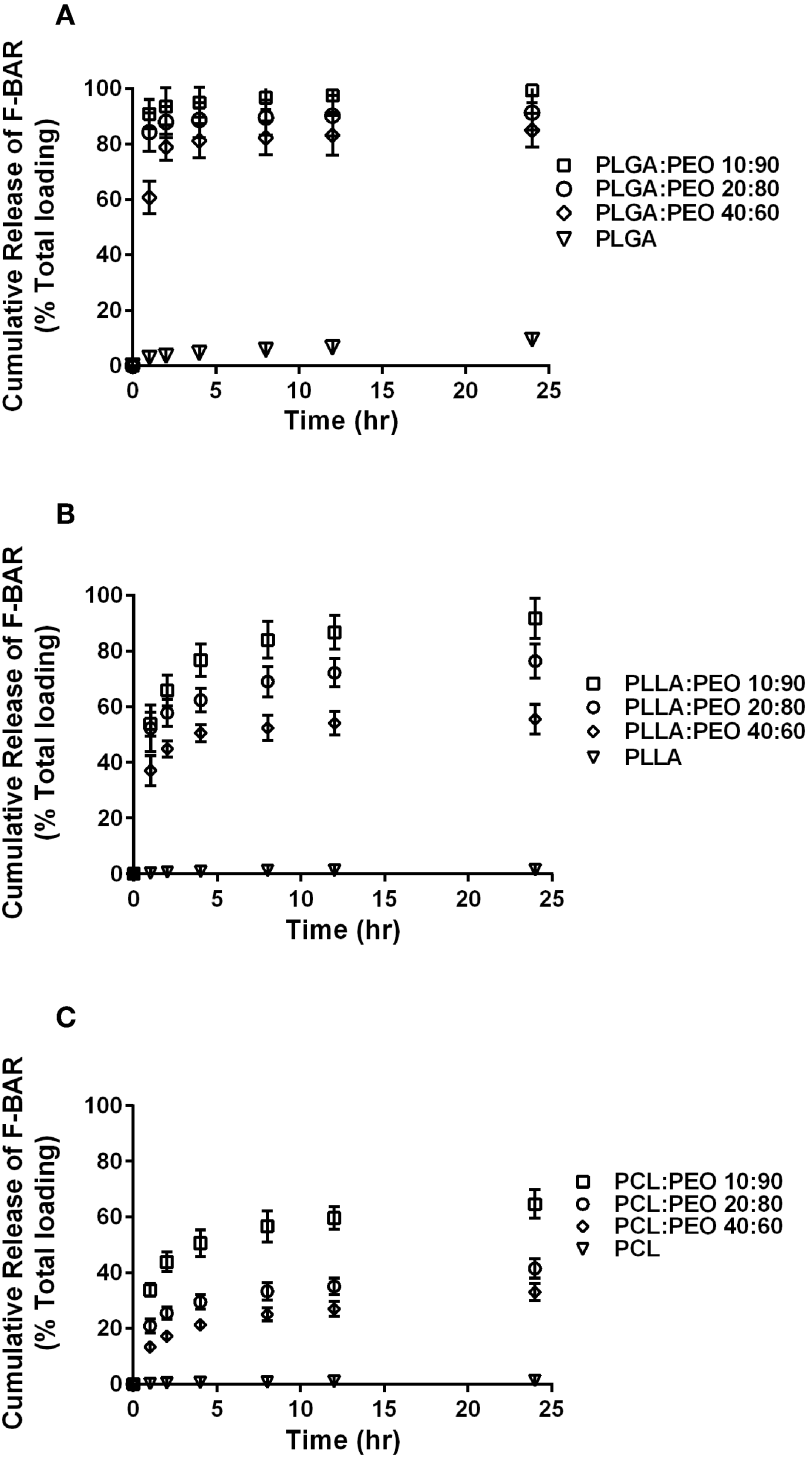
The cumulative release of F-BAR from the non-blended and PEO-blended formulations as a function of hydrophobic polymer type **(A)** PLGA, **(B)** PLLA, or **(C)** PCL and PEO ratio in each blend. The release of encapsulated BAR increases with an increase in PEO fraction. PLGA and PEO blends exhibit the most significant and rapid F-BAR release, relative to PLLA and PCL blends. For all polymer types, the 10:90 blends show the greatest release of BAR as compared to the 20:80 and 40:60 formulations at any given time point. PLGA:PEO (10:90) fibers provide the highest amount of BAR release across formulations. Data represent the mean ± standard deviation (*n* = 3) of three independent experiments.

The release of F-BAR from blended PLGA:PEO, PLLA:PEO, and PCL:PEO fibers with different blend ratios (40:60, 20:80, 10:90) as a function of hydrophobic polymer type, is shown in Figure 4. The importance of the PEO ratio in each hydrophobic fiber type, is emphasized in Figure 4, with the 10:90 formulation providing maximum release of F-BAR for each hydrophobic blend. Fibers comprised of 10:90 PLGA:PEO released 8.39 ± 0.06 μg BAR/mg EF, corresponding to 95 ± 0.26% of the incorporated F-BAR within the first 4 h, relative to PLLA:PEO and PCL:PEO 10:90 fibers with 76.8 ± 0.8% and 50.6 ± 0.8% of F-BAR release, respectively (Figures 4, 5). A significant reduction in the release of F-BAR was observed after 4 h for the 10:90 PLGA:PEO EFs and after 8 h for the other 10:90 PEO-blended formulations (Figure 4). For the 20:80 blended formulations, the PLGA:PEO fibers showed a maximum release of 88.7 ± 0.3%, compared to PLLA:PEO and PCL:PEO with 62.4 ± 2.1% and 29.6 ± 0.06% release, respectively, after 4 h. Similar trends in F-BAR release were observed for the 40:60 formulations with PLGA:PEO exhibiting the maximum release of 81.2 ± 0.1%, and PLLA:PEO and PCL:PEO releasing 50.6 ± 3.1% and 21.3 ± 0.2% after 4 h. Of the tested formulations, 40:60 PLGA:PEO, PLLA:PEO, and PCL:PEO released the least F-BAR within the first 4 h, and a significant reduction in release was observed after ~4 h for both the 20:80 and 40:60 formulations. Overall, the release trends for the different ratios of polymer blends were similar, with PLGA blends achieving the highest F-BAR release, followed by PLLA and PCL formulations.

### *P. gingivalis*/*S. gordonii* Biofilm Inhibition

**Figure 6:**
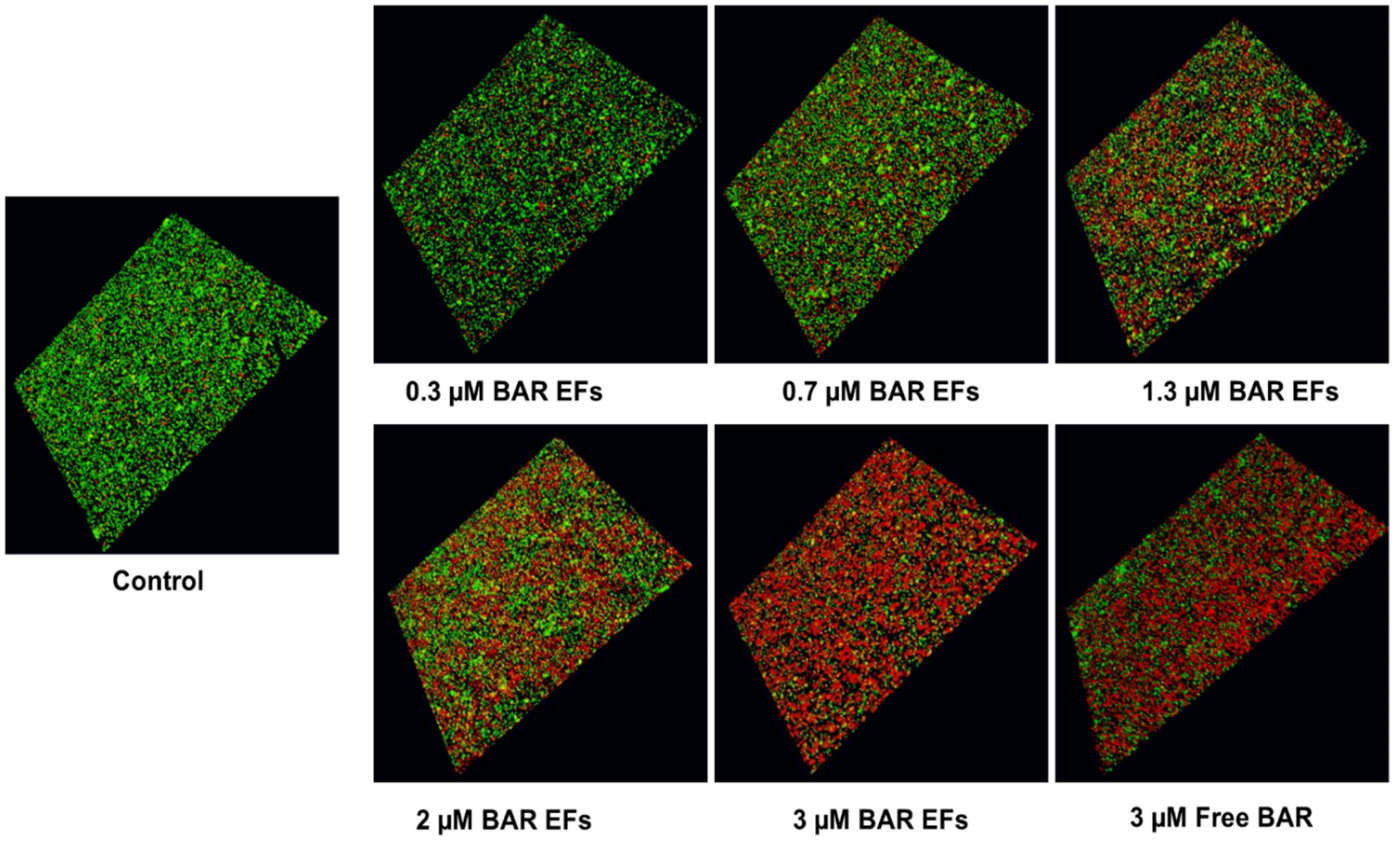
BAR-incorporated PLGA:PEO (10:90) EFs prevent *P. gingivalis* adherence to *S. gordonii*. Biofilms were visualized with confocal microscopy and the ratio of green (*P. gingivalis*) to red (*S. gordonii*) fluorescence in z-stack images was determined using Volocity image analysis software. Each grid represents 21 μm.

Given that the 10:90 PLGA:PEO blends achieved the highest release of F-BAR, the ability of the 10:90 PLGA:PEO BAR-EFs to inhibit or “prevent” *P. gingivalis* biofilm formation was assessed, relative to the administration of free BAR. To assess inhibition, 10:90 PLGA:PEO BAR-EFs or free BAR were administered to *P. gingivalis* for 24 h. Subsequently, BAR-EF or free BAR-treated *P. gingivalis* was incubated with immobilized *S. gordonii*. As shown in Figures 6, **8A**, *P. gingivalis* adherence was significantly reduced in the presence of 10:90 PLGA:PEO BAR-EFs. Biofilm formation was inhibited by 31, 42, or 82% by 0.3, 0.7, and 3.0 μM BAR-EFs, respectively. The maximum inhibition observed was similar to the 81% inhibition observed with free BAR (3 μM). BAR-incorporated EFs potently inhibited biofilm formation in a dose-dependent manner (IC50 = 1.3 μM). As expected, no statistical significance (*P* > 0.05) in inhibition was observed between BAR-incorporated EFs and free BAR.

### *P. gingivalis*/*S. gordonii* Biofilm Disruption

**Figure 7:**
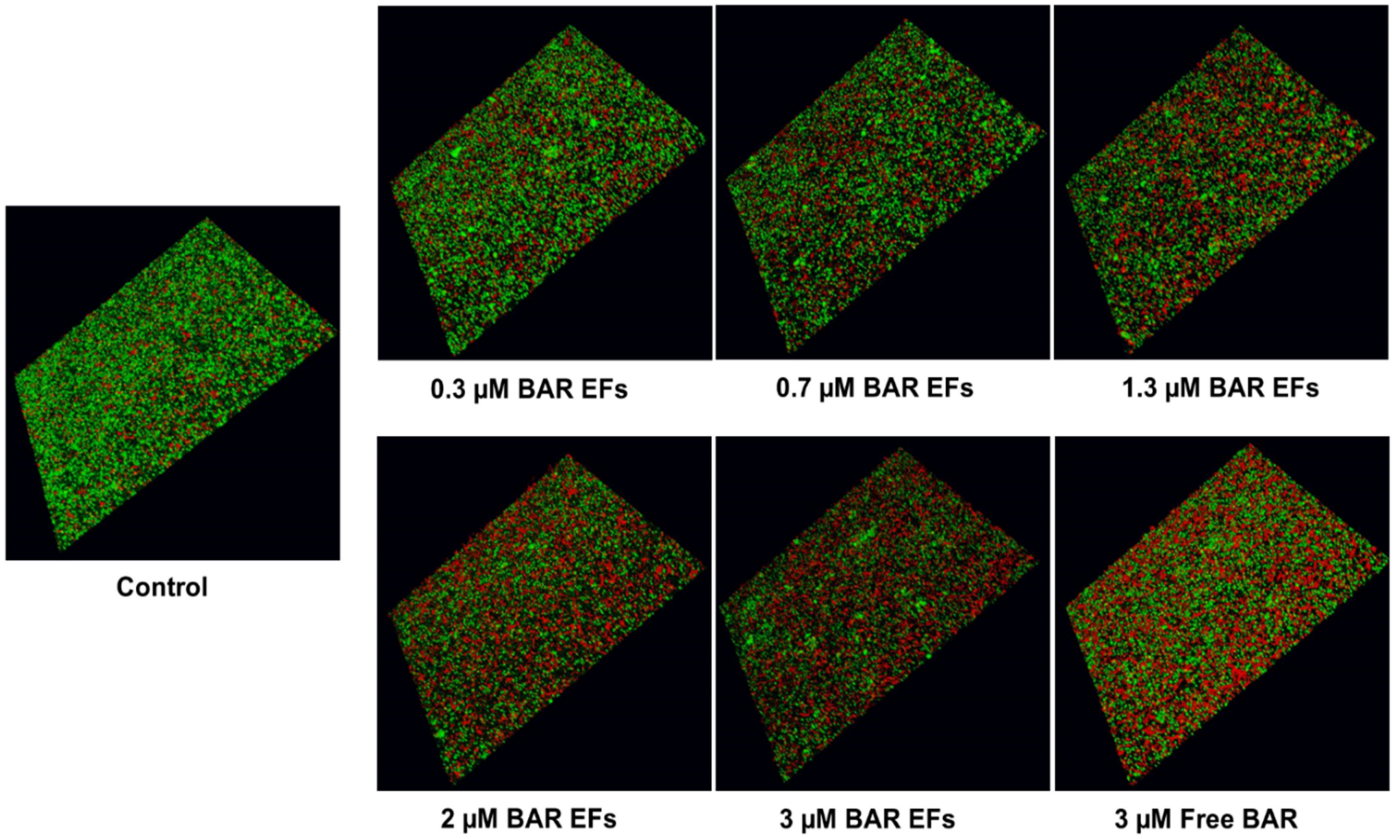
BAR-incorporated PLGA:PEO (10:90) EFs disrupt pre-established *P. gingivalis-S. gordonii* biofilms. Biofilms were visualized with confocal microscopy and the ratio of green (*P. gingivalis*) to red (*S. gordonii*) fluorescence in z-stack images was determined using Volocity image analysis software. Each grid represents 21 μm.

**Figure 8:**
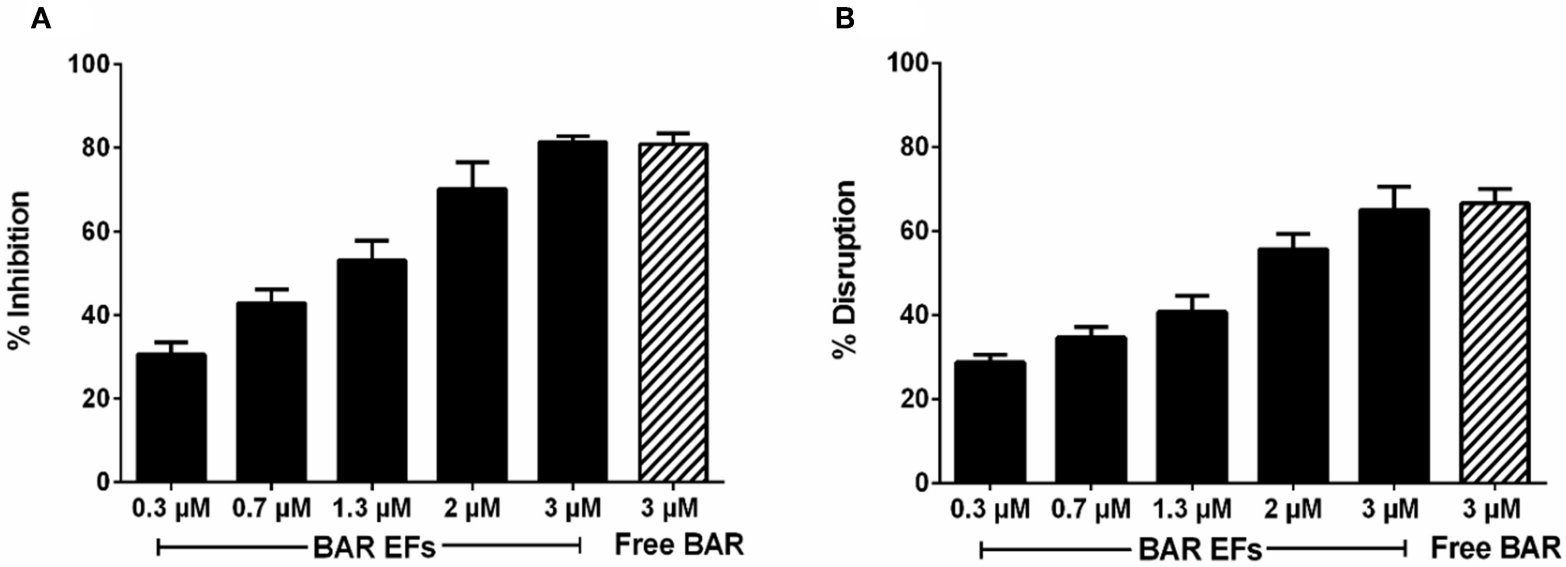
**(A)** Biofilm inhibition (prevention) and **(B)** disruption (treatment), as a function of different concentrations of BAR-incorporated PLGA:PEO (10:90) EFs and free BAR (3 μM). Data represent the mean ± standard deviation (*n* = 6) of six independent experiments.

The ability of the 10:90 PLGA:PEO BAR-incorporated EFs to disrupt or “treat” pre-existing *P. gingivalis/S. gordonii* biofilms was assessed (Figures 7, 8B). Dual-species biofilms were formed for 24 h, and were subsequently incubated for 3 h with BAR-incorporated EFs or free BAR. Biofilm formation was disrupted by 29, 34, or 66% by 0.3, 0.7, and 3.0 μM BAR-EFs. The maximum inhibition observed was similar to the 66% inhibition observed with free BAR (3 μM). Taken together, BAR-EFs exhibited effective biofilm disruption (IC50 = 2 μM) that was similar to free BAR (*P* > 0.05).

### Assessment of BAR and BAR-EFs *in vitro* Cytotoxicity

#### Hemolytic Assay

**Figure 9:**
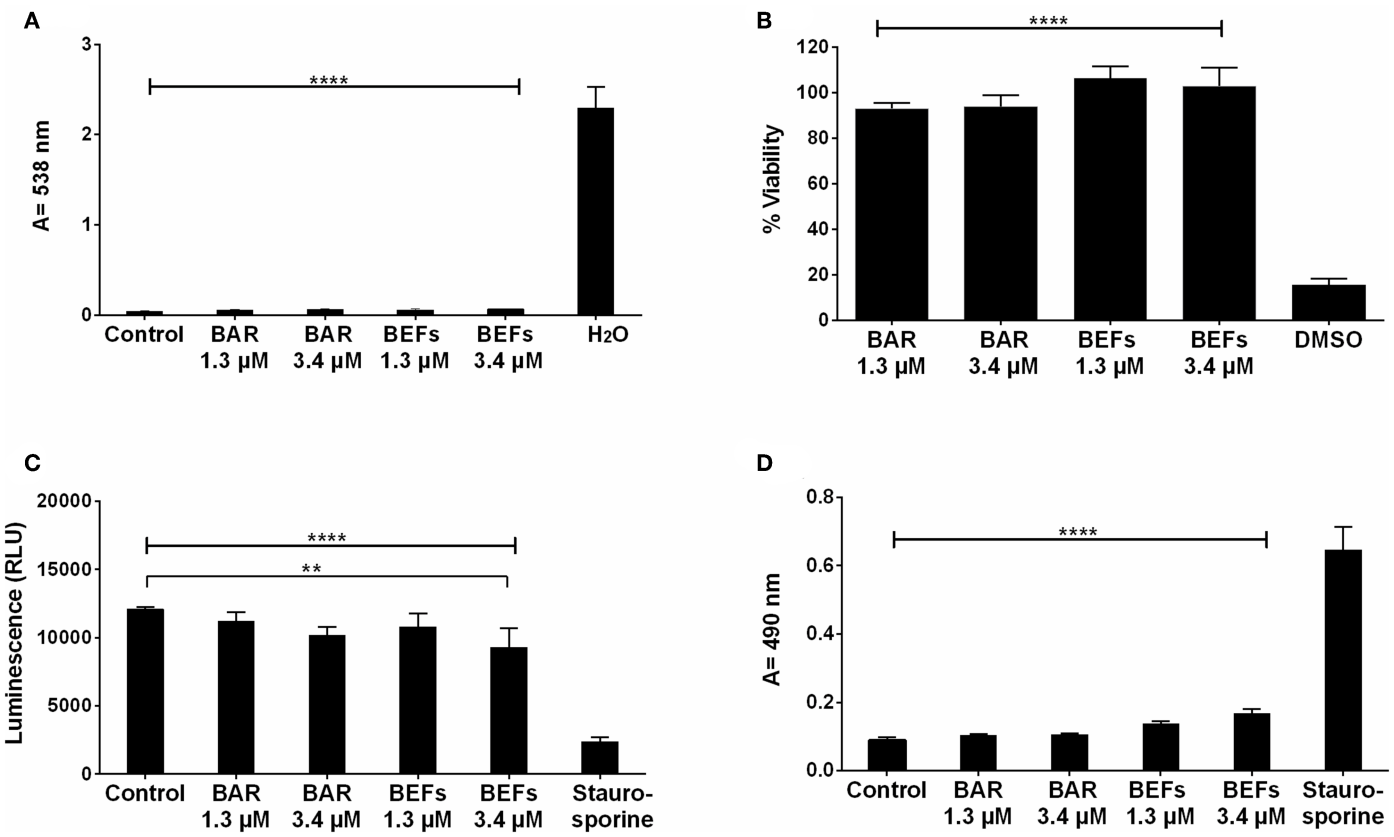
**(A)** The hemolytic activity of free BAR or 10:90 PLGA:PEO BAR-EFs (1.3, 3.4 μM) was assessed after administration to sheep erythrocytes for 3 h. Free BAR and BAR-EFs showed negligible hemolysis for sheep erythrocyte relative to release from H_2_O-treated cells (****P ≤ 0.0001). **(B)** The effect of free BAR and BAR-EFs (1.3, 3.4 μM) on TIGK cell viability was assessed. Free BAR and BAR-EFs were non-toxic, relative to cells treated with DMSO (****P ≤ 0.0001). **(C)** Metabolic activity of cells treated with free BAR or BAR-EFs (1.3, 3.4 μM) was assessed. BAR-EF (3.4 μM) treated cells showed decreases in ATP levels relative to medium-only treated cells, while TIGK cells treated with staurosporine demonstrated lower ATP levels than the cells treated with medium-only, free BAR, and BAR-EFs (**P ≤ 0.01,****P ≤ 0.0001). **(D)** TIGK cell membrane integrity was assessed after administration of free BAR or BAR-EFs (1.3, 3.4 μM) by measuring LDH release levels. None of free BAR or BAR-EF (1.3, 3.4 μM) treated cells released a significant level of LDH relative to medium-only treated cells. Staurosporine-treated cells demonstrated significantly elevated LDH levels (****P ≤ 0.0001). Data represent the mean ± standard deviation (*n* = 5) of five independent experiments.

The cytotoxicity of free BAR and 10:90 PLGA:PEO BAR-EFs was initially assessed by measuring the hemolytic activity against sheep red blood cells (RBCs). As shown in Figure 9A, neither free BAR nor BAR-EFs (1.3 or 3.4 μM BAR) induced hemolysis of RBCs.

#### MTT Assay

To determine the effect of free BAR or BAR-EFs on TIGK cell viability, cells were treated with free BAR or 10:90 PLGA:PEO BAR-EFs (1.3 or 3.4 μM) and viability was assessed using the MTT assay. As shown in Figure 9B, free BAR (1.3 or 3.4 μM) treated cells exhibited no loss in viability (*P* > 0.05), while BAR-EF (1.3 or 3.4 μM) treated cells showed higher viability (*P* ≤ 0.05), relative to medium-only treated cells.

#### ATP Assay

The metabolic activity of TIGK cells was assessed by measuring ATP levels. As shown in Figure 9C, cells treated with free BAR (1.3 or 3.4 μM) or 10:90 PLGA:PEO BAR-EFs (1.3 μM) showed no decrease in ATP relative to medium-only treated cells, while, cells treated with 10:90 PLGA:PEO BAR-EFs (3.4 μM) exhibited slightly lower levels of ATP relative to medium-only treated cells (9303.5 ± 1399 and 12094 ± 181 relative light units (RLUs), respectively, *P* ≤ 0.01). Staurosporine-treated cells demonstrated significantly lower levels of ATP (*P* ≤ 0.0001) than were observed for medium-only, free BAR, and 10:90 PLGA:PEO BAR-EF treated cells.

#### LDH Assay

Since some peptides are known to damage the cell membrane, LDH released in the cell media was evaluated as a marker for cell membrane integrity after free BAR or 10:90 PLGA:PEO BAR-EF treatment. Figure 9D shows that free BAR or BAR-EFs (1.3 or 3.4 μM) induced no change in levels of LDH released from cells, relative to medium-only treated cells. However, staurosporine induced a significantly higher level of LDH released from TIGK cells relative to cells treated with medium-only, free BAR, and BAR-EFs (*P* ≤ 0.0001).

#### Oxidative DNA Damage

**Figure 10:**
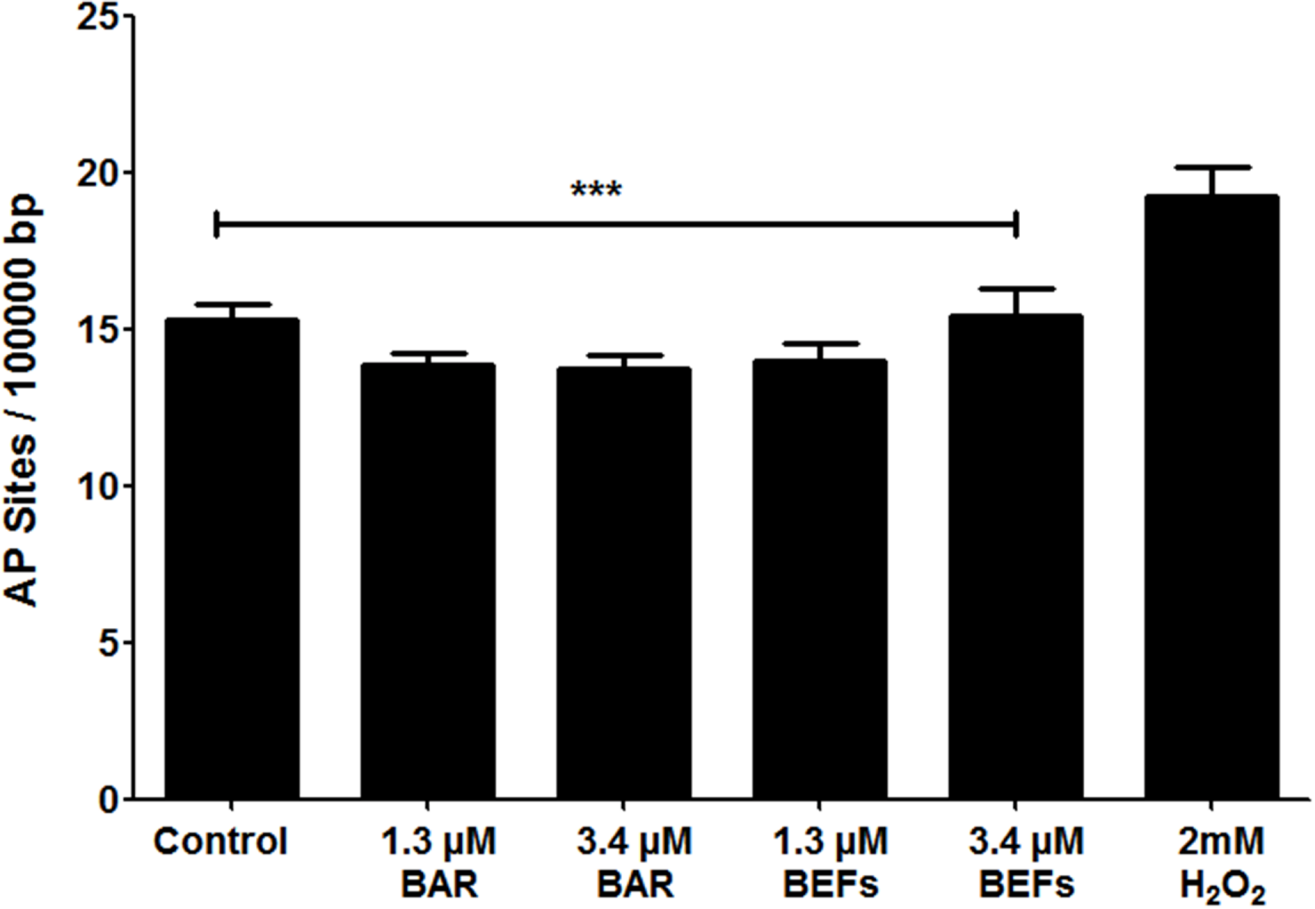
The number of AP sites per 100,000 base pairs of genomic DNA obtained from TIGK cells treated with free BAR or BAR-EFs (1.3, 3.4 μM). Negligible changes in the number of AP sites were observed in cells treated with free BAR or 10:90 PLGA:PEO BAR-EFs (1.3, 3.4 μM), relative to medium-only treated cells. However, TIGK cells treated with 2 mM H_2_O_2_ demonstrated significantly (***P ≤ 0.001) higher numbers of AP sites relative to the untreated control, free BAR and BAR-EF (1.3, 3.4 μM) treated cells. Data represent the mean ± standard deviation (*n* = 3) of three independent experiments.

The number of AP sites was determined as an oxidative stress marker for cells treated with free BAR or 10:90 PLGA:PEO BAR-EFs (1.3 or 3.4 μM). As shown in Figure 10, free BAR or BAR-EF treated (1.3 or 3.4 μM) cells demonstrated no change in the number of AP sites relative to medium-only treated cells, while cells treated with 2 mM H_2_O_2_ exhibited a significant increase in the number of AP sites relative to free BAR, BAR-EFs (1.3 or 3.4 μM), and medium-only treated cells (^***^P ≤ 0.001). These results suggested that neither free BAR nor BAR-EFs (1.3 or 3.4 μM) induce oxidative stress in TIGK cells.

## Discussion

One of the primary challenges facing the translation of active agents to clinical periodontitis therapy is the delivery and retention of efficacious concentrations of agent within the oral cavity. Local drug delivery vehicles in the form of films (Shifrovitch et al., 2009), strips (Friesen et al., 2002; Leung et al., 2005), and wafers (Bromberg et al., 2000) have been applied to periodontal disease, where the subgingival pockets act as a natural reservoir for these drug-loaded carriers. However, the methods used to fabricate these dosage forms include solvent casting, melt spinning and direct milling methods, which often prove to be labor intensive, time consuming, expensive, and potentially detrimental to the incorporation of more labile biological agents. Delivery platforms such as nanoparticles have also been investigated for both oral delivery and periodontitis applications by our group (Kalia et al., 2017; Mahmoud et al., 2018, 2019) and others (Napimoga et al., 2012; Seneviratne et al., 2014; Yao et al., 2014) to localize active agent delivery to the oral cavity (Napimoga et al., 2012; Seneviratne et al., 2014; Yao et al., 2014; Abou Neel et al., 2015; Kalia et al., 2017; Mahmoud et al., 2018, 2019). However, encapsulant release is less easily modulated, due to the large surface area and limited encapsulation efficiency (Gref et al., 1994; Kim et al., 2004). Moreover, nanoparticles may be “washed away” with saliva, resulting in lower retention in, and inadequate delivery to the oral cavity, necessitating increased doses to achieve efficacy.

In contrast, EFs, produced using a time and cost-efficient electrospinning process, can offer several advantages relative to other dosage forms. Some of these advantages include, the large surface-to-volume ratio of EFs, which can provide increased contact between the encapsulated bioactive molecule and the surrounding tissue, promoting release; high encapsulation efficiency, important for more labile biological molecules; mechanical stability and durability within the surrounding environment (Su et al., 2009); and importantly, the ability to tailor drug release, with for example, immediate, smooth, pulsatile, delayed, and biphasic release profiles (Sundararaj et al., 2013; Falde et al., 2015). In addition, fibers have the capacity to serve as a more durable delivery vehicle, providing enhanced retention and ensuring active agent release within the oral cavity, relative to the digestive tract. The durability of EFs may also offer a more convenient administration method, similar to films, but with the capability of providing prolonged release in desired applications. Given these attributes (Morie et al., 2016), we envisioned that designing EFs for administration to the oral cavity may provide a new dosage form in which to administer BAR, relative to the administration of free BAR, and a mechanism to improve therapeutic outcomes by increasing the localized concentration of, and in future work, retention of BAR. While we initially sought to test the capabilities of more rapid-release fibers for twice-daily applications, long-term we envision BAR-EFs may be administered similarly to rapid-release dental strips or as more slowly degradable implants to eliminate the need for surgical removal.

To date, polymeric EFs have been used in several biomedical applications including wound dressing materials (Liu et al., 2017), tissue regeneration (Inanç et al., 2009; Yang et al., 2009), and as drug delivery vehicles for bioactive molecules, antimicrobial agents (Chaturvedi et al., 2013), anti-inflammatory drugs (Batool et al., 2018), and anesthetics (Zafar et al., 2016). For periodontal applications, PLGA fibers have acted as cell scaffolds (Inanç et al., 2009), and have been combined with other polymers including PCL and PLA to deliver traditional antibiotics such as doxycycline (Chaturvedi et al., 2013) and metronidazole for the localized treatment of periodontitis (Zamani et al., 2010; Reise et al., 2012). However, hydrophobic-only fibers have exhibited delivery limitations such as poor wettability, combined with inadequate flexibility and stiffness properties. Despite this, these and other more biodegradable fiber types such as polydioxanone and PLA:PCL/gelatin fibers incorporating ciproflaxin and tetracycline respectively, have significantly inhibited periodontal pathogens (Bottino et al., 2014; Shahi et al., 2017). However, these fiber types have focused on the delivery of non-specific antibiotics that may have adverse effects on healthy microbiota in the oral cavity.

Given this delivery potential combined with our observed specific targeting with BAR peptide (Kalia et al., 2017; Mahmoud et al., 2018, 2019), we sought to fabricate and compare non-blended (hydrophobic-only polymeric fibers) with blended BAR-incorporated EFs using a uniaxial electrospinning approach. We initially formulated 1% w/w (BAR/polymer) EFs, resulting in a theoretical loading of 10 μg BAR per mg of polymer, a concentration shown in our previous work to inhibit biofilm formation. All resulting EFs demonstrated high F-BAR loading; however, the release kinetics of the non-blended PLGA, PLLA, and PCL fibers revealed minimal release of F-BAR over 24 h. We attributed the high hydrophobicity of the non-blended PLGA, PLLA, and PCL fibers to minimal eluate penetration past the outermost fiber layer. Moreover, hydrophobic sequences in BAR peptide may promote hydrophobic interactions with the purely hydrophobic non-blended fibers, resulting in lower release.

While hydrophobic polymers have been used in numerous applications outside of the oral cavity, to obtain time frames of release relevant to oral delivery (here, twice-daily), we sought to modulate fiber hydrophobicity with the addition of hydrophilic PEO in ratios (PLGA/PLA/PCL:PEO 40:60, 20:80, and 10:90). In addition, varying of EFs processing variables and type of materials resulted in diameter, porosity, and morphology changes that can regulate the drug profile (Huang et al., 2006). Previous work has shown that blending hydrophobic polymers with more hydrophilic polymers increases the release of biological molecules such as lysozyme, while maintaining protein activity (Li et al., 2008). In addition, many studies have shown that the addition of PEO to protein solutions can improve protein stability (Casper et al., 2005; Li et al., 2006, 2008) and increase mucoadhesivity (Choi et al., 2014), which may help to increase retention in future applications. Moreover, recent work has demonstrated that the incorporation of PEO within hydrophobic fibers increases pore formation and fiber weight loss, prompting more rapid degradation relative to non-blended fibers (Kim et al., 2007; Evrova et al., 2016).

In agreement with other studies (Evrova et al., 2016), increasing the fiber hydrophilicity with the addition of PEO significantly improved BAR release from the blended fibers. Moreover, the incorporation of PEO enabled rapid degradation, eliminating the need for fiber removal after administration. Last, while hydrophilic molecules have been shown to have more affinity to and miscibility with PEO (enabling release), we postulate that the electrospinning process itself can impact encapsulant location, particularly within hydrophobic:hydrophilic blended fibers, prompting variable release kinetics. That is, electrospinning may promote F-BAR aggregation near the fiber surface, due to charge repulsion (Szentivanyi et al., 2011), contributing to the burst release observed in all blended fibers. Among the hydrophobic polymers, PLGA EFs demonstrated the highest release at early time points, followed by PLLA and PCL formulations. We propose that the increased release of BAR from PLGA EFs is attributed to its amorphous and less hydrophobic properties, relative to the more hydrophobic PLLA and PCL polymers, while PCL:PEO EFs demonstrated the least release due to their crystalline and slightly more hydrophobic characteristics.

Although EFs demonstrated high encapsulation efficiency, potently inhibited biofilm formation, and disrupted pre-existing biofilms, they demonstrated burst release within 4 h and minimal release thereafter. To achieve the IC50 of BAR (4 μg/mL) over a duration of 12 h, we acknowledge that loading capacity and release must be increased and optimized, respectively. The fibers fabricated in this study were formulated with 1% w/w BAR/polymer, as previous work has shown that fibers fabricated with a theoretical loading higher than 1% w/w, using a uniaxial blended spinning process, may result in significant initial burst release (Kim et al., 2004). In future work, techniques like co-axial or emulsion electrospinning may be adopted to increase loading and optimize release kinetics while maintaining peptide stability (Li et al., 2010; Sebe et al., 2015). Several studies have used co-axial electrospinning to sustain the release and maintain the bioactivity of biological molecules since aqueous solutions and solvents can remain separate during the electrospinning process (Ji et al., 2010). Alternatively, emulsion electrospinning may help to encapsulate hydrophilic agents within the core, thereby providing more sustained and incremental release (Li et al., 2010).

In addition to achieving delivery and retention within the oral cavity, the penetration and disruption of complex biofilms is an obstacle to delivery, which is not fully reflected in *in vitro* models. Achieving high localized concentrations within the oral cavity has traditionally required the administration of elevated doses, which may induce adverse effects (van Winkelhoff et al., 2000). However, a delivery system, such as fibers, that can facilitate ease of administration, while localizing doses applied directly to the subgingival pocket or as topically administered dental strips, may be one option to improve efficacy and overcome toxicity challenges associated with high dose and repeated administrations. To meet these needs, both non-degradable and lesser studied degradable vehicles have been developed for the delivery of antibiotics as primary or adjunct therapies and some are commercially available (Friesen et al., 2002; Kenawy el et al., 2002; Ahuja et al., 2003; Aimetti et al., 2004; Kim et al., 2004; Zamani et al., 2010; Batool et al., 2018). However, the utilization of non-biodegradable vehicles requires surgical removal, which can increase the risk of infection and foreign body response (Vyas et al., 2000). Conversely, a degradable delivery platform, such as those described here, for intra-pocket or topical administration may eliminate the need for removal and avoid the risk of associated infection and immune response.

In addition to minimizing surgery and/or surgical intervention, the specific localization or “targeting” of active agents and delivery vehicles, can enhance their efficacy against periodontal diseases and decrease adverse effects (Maze et al., 1996). Many broadly active mucoadhesive materials such as carboxymethyl cellulose (CMC) (Cai et al., 2017), polyacrylic acid (Carbopol) (Kilicarslan et al., 2014); polyethylene glycol (PEG) (Endo et al., 2013) and polyvinyl alcohol (PVA) (Nafee et al., 2003) have been integrated to improve agent adhesion and retention in the oral cavity; however, these are non-specific approaches that facilitate adhesion to all oral tissue. An alternative approach is to exploit specific protein-protein interactions that drive interspecies coaggregation between oral organisms that promote adhesion and infection within specific niches in the oral cavity. One of these proteins, coaggregation factor A (CafA) (Reardon-Robinson et al., 2014), is known to facilitate *Actinomyces*/*Streptococcus* coaggregation in the oral cavity (Reardon-Robinson et al., 2014). Thus one strategy to improve BAR-EF delivery and retention in future work is to surface-modify BAR-EFs with CafA for targeting to streptococcal cells. These advancements may be helpful in achieving dual-targeting via BAR and CafA as intra-pocket delivery systems, in which fibers can be immobilized in the subgingival pocket for a longer duration. Thus in ongoing work, we are focused on the development of targeted EFs to enhance retention and adherence to specific bacterial targets in the oral cavity. Ultimately, advanced formulations that extend release and target keystone species via adhesion may be applied once- or twice-daily to localize BAR release and exert enhanced prophylactic effect in the oral cavity without the need to remove the fibers after application.

## Conclusions

Taken together, these results demonstrate the feasibility, versatility, and straightforward approach of electrospinning EFs that release therapeutically-relevant concentrations of BAR, to specifically target periodontal pathogens. In our studies, fibers with increasing PEO content significantly enhanced F-BAR release, while the most promising 10:90 PLGA:PEO formulation provided 95% F-BAR release after 4 h, inhibited biofilm formation in a dose-dependent manner (IC50 = 1.3 μM), and efficiently disrupted dual-species biofilms (IC50 = 2 μM). Our results suggest that BAR-incorporated EFs may provide an alternative and specifically-targeted rapid-release platform to inhibit and disrupt dual-species biofilms, that we envision may be applied twice-daily to exert prophylactic effect in the oral cavity without the need to remove the fibers after application. Future studies will be focused on optimizing the release kinetics of BAR from blended EFs for more sustained durations of 12–24 h, by utilizing altered fabrication procedures like emulsion and co-axial electrospinning (Li et al., 2010; Sebe et al., 2015).

## Data Availability Statement

The datasets generated for this study are available on request to the corresponding author.

## Author Contributions

MM and SS performed the experiments, statistical analysis, and drafted the manuscript. KC imaged electrospun fibers by using scanning electron microscopy. JS-R conceived of the study, participated in its design and coordination, reviewed experimental data, and drafted the manuscript. DD conceived of the study, participated in its design and coordination, reviewed experimental data, and drafted the manuscript. All authors read and approved the final manuscript.

### Conflict of Interest

The authors declare that the research was conducted in the absence of any commercial or financial relationships that could be construed as a potential conflict of interest.
